# Identification of a homozygous frameshift mutation in the *FGF3* gene in a consanguineous Iranian family: First report of labyrinthine aplasia, microtia, and microdontia syndrome in Iran and literature review

**DOI:** 10.1002/mgg3.2168

**Published:** 2023-03-19

**Authors:** Fereshteh Jamshidi, Ebrahim Shokouhian, Marzieh Mohseni, Kimia Kahrizi, Hossein Najmabadi, Mojgan Babanejad

**Affiliations:** ^1^ Genetics Research Center University of Social Welfare and Rehabilitation Sciences Koodakyar Alley, Daneshjoo Blvd., Evin St. 1985713834 Tehran Iran

**Keywords:** *FGF3*, Iran, LAMM syndrome, whole‐exome sequencing

## Abstract

**Background:**

To date, over 400 syndromes with hearing impairment have been identified which altogether constitute almost 30% of hereditary hearing loss (HL) cases around the globe. Manifested as complete or partial labyrinthine aplasia (severe malformations of the inner ear structure), type I microtia (smaller outer ear with shortened auricles), and microdontia (small and widely spaced teeth), labyrinthine aplasia, microtia, and microdontia (LAMM) syndrome (OMIM 610706) is an extremely rare autosomal recessive condition caused by bi‐allelic mutations in the *FGF3* gene.

**Methods:**

Using the whole‐exome sequencing (WES) data of the proband, we analyzed a consanguineous Iranian family with three affected members presenting with congenital bilateral HL, type I microtia, and microdontia.

**Results:**

We discovered the homozygous deletion c.45delC in the first exon of the *FGF3* gene, overlapping a 38.72 Mb homozygosity region in chromosome 11. Further investigations using Sanger sequencing revealed that this variant co‐segregated with the phenotype observed in the family.

**Conclusion:**

Here, we report the first identified case of LAMM syndrome in Iran, and by identifying a frameshift variant in the first exon of the *FGF3* gene, our result will help better clarify the phenotype–genotype relation of LAMM syndrome.

## Graphical abstract

Characterized by bilateral congenital deafness, severe malformations of the inner ear (Michel's aplasia), shortened upper half of auricles (microtia type I), and small and widely spaced teeth (microdontia), labyrinthine aplasia, microtia, and microdontia (LAMM) syndrome is a very rare disorder that has been reported in more than 20 families worldwide. Here, we report the first identified case of LAMM syndrome in Iran, and by identifying a frameshift variant in the first exon of the FGF3 gene, our result will help better clarify the phenotype–genotype relation of LAMM syndrome.

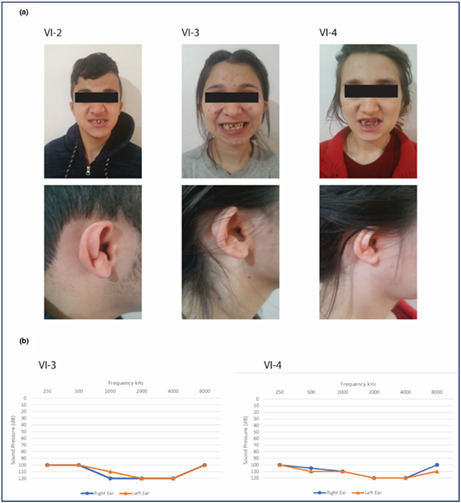

## 1 INTRODUCTION

Hearing loss (HL) is the most common sensorineural disorder, affecting one in every 500 individuals. Screening data from developed countries show that almost 50% of all HL cases are genetic, of which almost 30% are syndromic. To date, more than 400 syndromes with hearing impairment have been identified (Sheffield & Smith, 2019). Moreover, an identifiable inner ear malformation can be found in 15%–20% of cases with severe or profound sensorineural HL (Joshi et al., 2012).

Accounting for 1% of the inner ear malformations, complete labyrinthine aplasia (CLA) was first described by Michel in 1863, and is characterized by the complete absence of the inner ear structure (Ozgen et al., 2009). In 1991, Hersh et al. reported a 2.5‐year‐old female with congenital sensorineural deafness due to Michel's aplasia. Moreover, the patient was presented with type I microtia (small auricles with or without malformation), and microdontia (small and widely spaced teeth). However, it was until 2007 that Tekin et al. (2007) discovered homozygous mutations in the fibroblast growth factor 3 (*FGF3*) gene could lead to labyrinthine aplasia, microtia, and microdontia (LAMM) syndrome (labyrinthine aplasia, microtia, and microdontia—OMIM 610706). LAMM syndrome is an extremely rare autosomal recessive condition, for which no prevalence has yet been established (Ordonez & Tekin, 1993). The three main phenotypes of the LAMM syndrome include, complete or partial absence of the inner ear structures (Michel's aplasia), type I microtia with or without large skin tags on the upper sides of the auricles, and microdontia.

The advent of the new high‐throughput sequencing technologies has been of great help for the identification of novel variants. Various next‐generation sequencing methods, including whole‐exome and whole‐genome sequencing, have been used to identify novel variations and genes associated with HL (19, 20). Studies have shown that more than 89% of the reported pathogenic variants in the ClinVar database (https://www.ncbi.nlm.nih.gov/clinvar/) are located in the coding region of the genome (21). Therefore, considering the high cost and the overwhelming amount of data generated by the whole‐genome sequencing (WGS), in most cases whole‐exome sequencing (WES) is the more reasonable approach for clinical and research purposes.

To date, 23 pathogenic and likely pathogenic variants in the *FGF3* gene have been reported to be associated with LAMM syndrome in PubMed, Web of Science, ClinVar, and HGMD databases (Table 1). Here we add to the pathogenic allelic heterogeneity of *FGF3* by implicating one novel pathogenic variant in *FGF3*, which segregates with LAMM syndrome in an Iranian family. This study is the first reported case of LAMM syndrome in Iran and will provide further insight into the molecular biology of LAMM syndrome.

**TABLE 1:** Mutations found in *FGF3* gene and their related clinical features.

Mutation	Reference SNP	Zygosity	Hearing evaluation	Inner ears	Outer ears	Teeth	Other clinical features	Reference
c.17 T > C	rs121917706	Homozygote	Congenital bilateral profound deafness	Complete labyrinthine aplasia	Microtia type I, Anteverted ears, Bilateral large skin tags	Microdontia	Stenosis of jugular foramen, enlarged emissary vein	Tekin et al. (2008)
c.45delC	rs1434810965	Homozygote	Congenital bilateral profound deafness	Complete labyrinthine aplasia	Microtia type I, Anteverted ears	Microdontia with thin enamel	NA	This study
c.146A > G, c.310C > T	rs281860300	Compound Heterozygote	Congenital bilateral profound deafness	Complete labyrinthine aplasia	Microtia type I with anteverted ears	Microdontia	NA	Sensi et al. (2011)
c.150C > A	rs281860301	Homozygote	Congenital bilateral profound deafness	Complete labyrinthine aplasia	Microtia type I with Protruding ears	Microdontia, oligodontia	NA	Dill et al. (2011)
c.166C > T	rs782324453	Homozygote	Congenital bilateral profound deafness	NA	Microtia type I	Microdontia	NA	Doll et al. (2020)
c.173 T > C, c.283C > T	NA	Compound Heterozygote	Congenital bilateral profound deafness	Complete labyrinthine aplasia	Microtia type I with preauricular skin tags	Microdontia	NA	al Yassin et al. (2019)
c.196G > A	rs121917705	Homozygote	Congenital bilateral profound deafness	Complete labyrinthine aplasia	Microtia type I, unilateral skin tags	Microdontia, oligodontia, thin enamel	Prontocerebral arachnoid cyst	Basdemirci et al. (2019)
c.196G > T	rs121917705	Homozygote	Congenital bilateral profound deafness	Complete labyrinthine aplasia	Microtia type I with anteverted ears and asymmetrical dysplastic ears	Microdontia	NA	Alsmadi et al. (2009)
c.254delT	rs281860302	Homozygote	Congenital bilateral profound deafness	Complete labyrinthine aplasia	Microtia type I with anteverted ears and large skin tags	Microdontia	NA	Tekin et al. (2008)
c.255del	rs281860302	NA	NA	NA	NA	NA	NA	ClinVar
c.270dup	rs1554981083	NA	NA	NA	NA	NA	NA	ClinVar
c.283C > T	rs281860303	Homozygote	Congenital bilateral profound deafness	Complete labyrinthine aplasia	Variable, microtia type I	Variable, microdontia	Hypoplastic deformed petrous pyramids, large arachnoid cyst	al Yassin et al. (2019); Ramsebner et al. (2010); Riazuddin et al. (2011)
c.284G > A, c.534C > G	rs558206333	Compound Heterozygote	Congenital bilateral profound deafness	Cystic cochlea‐vestibular malformation	Protruding ears without microtia	Microdontia	Hypoplastic eighth cranial nerve	al Yassin et al. (2019)
c.310dup	NA	NA	NA	NA	NA	NA	NA	ClinVar
c.310C > T	rs121917704	Homozygote	Congenital bilateral profound deafness	Complete labyrinthine aplasia	Microtia type I with anteverted ears	Microdontia	Delayed motor skills, absence of cochleovestibular nerve, subarachnoid cyst	Riazuddin et al. (2011); Tekin et al. (2007)
c.317A > G, c.457_458del	rs281860307	Compound Heterozygote	Congenital bilateral profound deafness	Complete labyrinthine aplasia	Microtia type I	Microdontia	Hypoplastic petrous pyramid	Sensi et al. (2011)
c.325_327delinsA	NA	Homozygote	Congenital bilateral profound deafness	Complete labyrinthine aplasia	Microtia type I	Microdontia	Bilateral absence of the eighth cranial nerve	al Yassin et al. (2019)
c.394del	rs281860304	Homozygote	Congenital bilateral profound deafness	Complete labyrinthine aplasia	Microtia type I	Microdontia	Absence of cochleovestibular nerve, subarachnoid cyst	Riazuddin et al. (2011)
c.462C > G	rs782712529	NA	NA	NA	NA	NA	NA	ClinVar
c.466 T > C	rs121917703	Homozygote	Congenital bilateral profound deafness	Complete labyrinthine aplasia	Microtia type I	Microdontia with the loss of tooth height	Gross motor skills during infancy due to impaired balance	Tekin et al. (2007)
c.534C > G	rs782081344	Homozygote	Congenital bilateral profound deafness	Complete labyrinthine aplasia	Microtia type I, Anteverted ears, Bilateral large skin tags	Microdontia	Bilateral absence of the eighth cranial nerve, Hypophosphatemic rickets	al Yassin et al. (2019); Lallar et al. (2021); Singh et al. (2014)
c.616del	rs281860305	Homozygote	Congenital bilateral profound deafness	Complete labyrinthine aplasia	Microtia type I	Microdontia with the loss of tooth height	Bilateral absence of cochleovestibular nerve, strabismus	Tekin et al. (2007)
c.625C > T	rs374453035	NA	NA	NA	NA	NA	NA	ClinVar

## 2 MATERIALS AND METHODS

### 2.1 Study participants

An Iranian family with Azeri ethnicity was first referred to the Genetics Research Center (GRC) at the University of Social Welfare and Rehabilitation Sciences (USWR) due to congenital HL. Affected members underwent complete physical examination, and hearing thresholds were measured using standard protocols by puretone audiometry at 250, 500, 1000, 2000, 4000, and 8000 Hz. Written consent forms were obtained from all members of the family, and whole blood samples were collected, from which genomic DNA was extracted using routine methods. Subsequently, the proband underwent WES. The procedure used in this study has been approved and reviewed by the USWR ethics committee.

### 2.2 Next‐generation sequencing

Agilent SureSelectXT Human All Exon V6 (Agilent Technologies Inc, Santa Clara, CA, USA) was used for exome enrichment, and the Illumina NextSeq500 (Illumina, San Diego, California, USA) was used to sequence the libraries with paired‐ended read lengths of 100 bp. Quality control analysis was performed using the FastQC toolkit, and sequences were mapped to the UCSC hg19 human reference genome using the Burrows‐Wheeler Aligner (BWA) (Li & Durbin, 2010). The Genome Analysis Toolkit version 4.0 (GATK4.0, Broad Institute, Cambridge, MA, USA) was applied for BAM processing, realignment, base quality recalibration, and variant calling (Van der Auwera et al., 2013). The resulting variant calling format (VCF) file was annotated using ANNOVAR (Wang et al., 2010). Variants were then filtered according to their quality (depth > 3, quality score > 30) and minor allele frequency (MAF < 0.01). Variant prioritization was based on variant‐type, conservation (GERP and phyloP), and the scores from in silico prediction tools, including SIFT (Sim et al., 2012), PolyPhen2 (Adzhubei et al., 2010), and the Combined Annotation Dependent Depletion (CADD) (Rentzsch et al., 2021).

### 2.3 Autozygosity mapping

Due to the consanguineous nature of the family, we used the Automap tool (Quinodoz et al., 2021) to identify the runs of homozygosity (ROH) by using the WES VCF file as input. The analysis was conducted by using the tool's default settings, and after receiving the output files, variants identified by WES were further filtered according to the ROH regions.

### 2.4 Segregation analysis

Sanger sequencing and PCR primers were designed using the web‐based tool, Pimer3 (https://primer3.ut.ee/). The primers used in this study include the forward 5′‐CCCACCTTTCCCGCGAAG‐3′, and the reverse 5′‐CTCACTGTAGGCGCTGTTCT‐3′. The PCR products were sequenced on ABI 3130 Sequencer, and the chromatograms were compared to the genomic sequence of the *FGF3* (NM_005247.4) and changes were confirmed using the CodonCode Aligner software.

## 3 RESULTS

### 3.1 Clinical presentation

**FIGURE 1:**
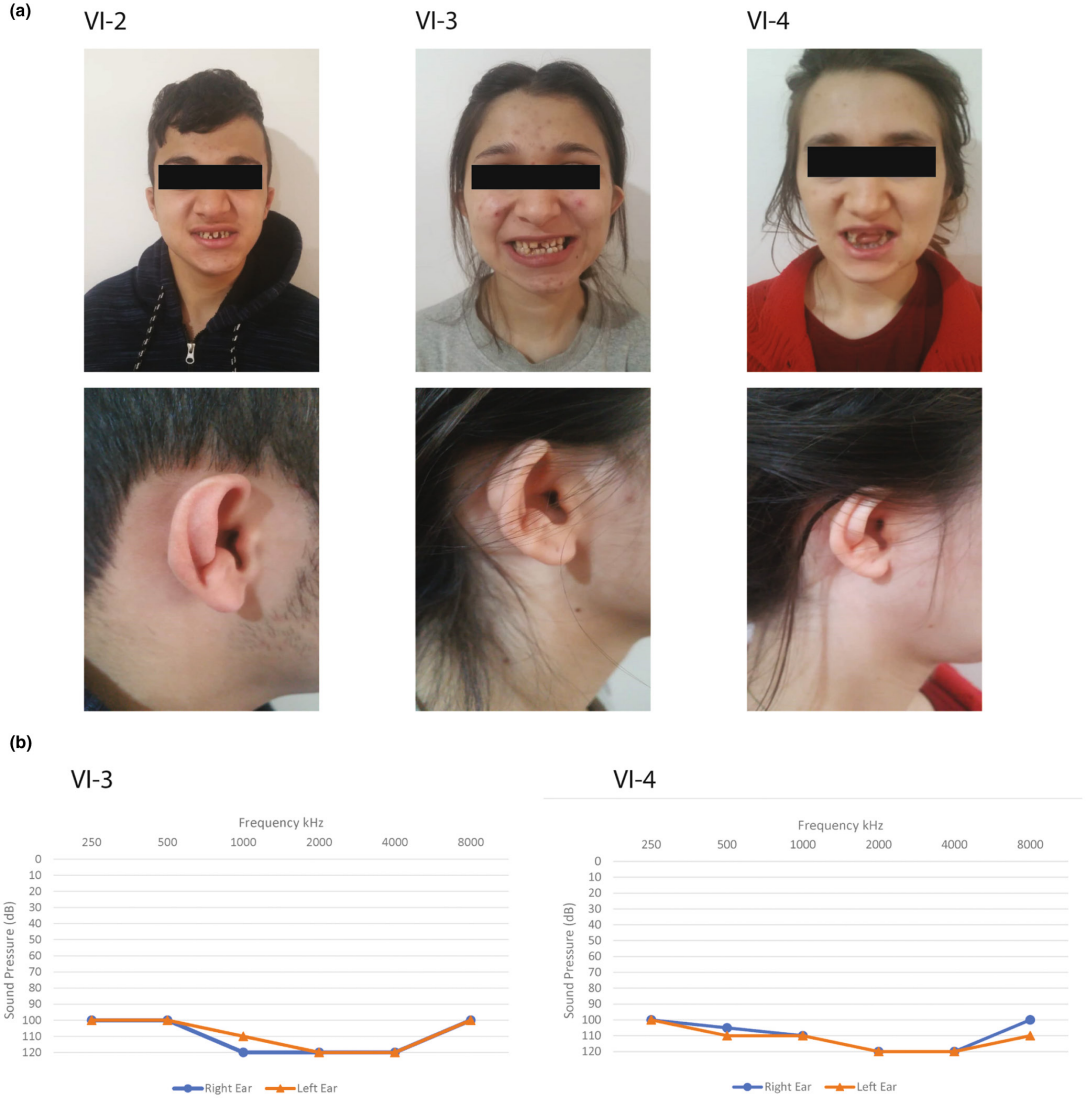
Pictures showing the facial characteristics of three Iranian siblings diagnosed with labyrinthine aplasia, microtia, and microdontia syndrome, and their respective audiograms. Note the microdontia with widely spaced teeth and microtia with shortening of the upper third of the auricles apparent in all of the affected individuals (a). Pure tone audiometry with frequencies 250 Hz to 8000 was applied to all family members. The resulting audiograms consistently show profound hearing loss in all affected members (b).

**FIGURE 2:**
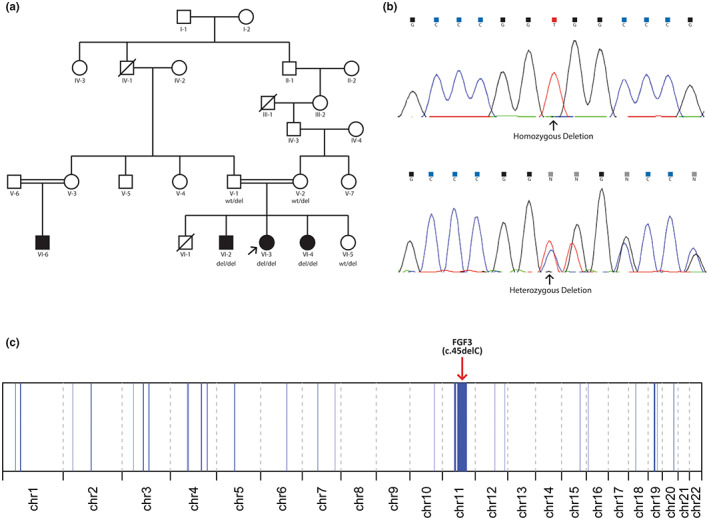
This picture shows the pedigree of a consanguineous Iranian family with labyrinthine aplasia, microtia, and microdontia syndrome (a), and the homozygous and heterozygous mutations found in the affected and healthy members respectively (b). Using the Automap software, autozygosity mapping was conducted for the autosomal chromosomes, and as marked, the mutation in the FGF3 gene was found in a large stretch of a homozygosity region (38.72 Mb) in chromosome 11 (c).

A consanguineous Iranian family with Azeri ethnicity and three affected children, one male and two females (Figure 2a), born from healthy parents was entered into the study. The main complaint for which they were referred to the USWR genetics research center was bilateral severe to profound congenital HL, diagnosed at birth (Figure 1b). After further physical examinations, it was noticed that all affected members were presented with shortening of the upper third of the auricles, prominent and bifurcated antihelix ears (compatible with microtia type I), and small and widely spaced teeth with loss of tooth height and thin enamel (compatible with microdontia) (Figure 1a). The parents and the healthy sister did not have any of the LAMM syndrome manifestations, meaning that the results from hearing evaluation of the healthy individuals were in normal ranges and no tooth or outer ear malformations were observed.

### 3.2 Genetic diagnosis

Variant calling from the WES data revealed 377,040 variants in total, of which only 5094 remained after filtering based on MAF and variant quality. In addition, autozygosity mapping revealed 95.26 Mb of homozygosity regions throughout autosomal chromosomes, including a 38.72 Mb region in chromosome 11 which overlapped 11 rare homozygous exonic variants (Figure 2c). Of particular interest was the homozygous frameshift deletion in exon one of the *FGF3* gene, c.45delC (Figure 2b). This variant has only one allele count in the gnomAD v2.1.1 database, and it is located on the first exon of the gene. The c.45delC variant is predicted to create a premature stop codon 63 amino acids downstream of the deletion, causing the transcript to undergo nonsense‐mediated decay (predicted based on the 50‐nt rule). Moreover, this variant was confirmed using Sanger sequencing, and it co‐segregates with LAMM syndrome in the family. Therefore, based on the American College of Medical Genetics guidelines, using the PVS1, PM2, and PP1 scores, this variant can be classified as pathogenic.

## 4 DISCUSSION

Characterized by bilateral congenital deafness, severe malformations of the inner ear (Michel's aplasia), shortened upper half of auricles (microtia type I), and small and widely spaced teeth (microdontia), LAMM syndrome is a very rare disorder that has been reported in more than 20 families worldwide (Ordonez & Tekin, 1993). In addition to the four main symptoms, motor development delay during infancy (probably due to impaired balance), large skin tags on the upper side of the auricles, malformation of the middle ear, stenosis of the jugular foramen, enlarged emissary veins, presence of intracranial arachnoid cyst, and absence or narrowing of the eighth cranial nerve have been observed in affected individuals (Ordonez & Tekin, 1993; Riazuddin et al., 2011; Tekin et al., 2008). In addition, in some cases unique phenotypic features were also diagnosed, including reduced kidney size, hypophosphatemic rickets, and type I diabetes (Al Yassin et al., 2019; Singh et al., 2014). However, it is possible that these features are due to variants in other genes or environmental factors.

The genetic etiology of LAMM syndrome was first identified in 2007 when Tekin et al. (2007) showed that mutations in the fibroblast growth factor 3 gene (FGF3 – OMIM 164950) co‐segregated with the phenotype in the affected families, and so far, 23 pathogenic or likely pathogenic variants have been reported in ClinVar, HGMD, PubMed, and Web of Science databases. The *FGF3* gene (also known as INT2 and HBGF‐3), mapped to chromosome 11q13.3, contains three exons and encodes a 239‐amino‐acid‐long protein. Studies in mice have shown that the loss of function of the FGF3 protein leads to a malformed inner ear structure with dilated or absent semi‐circular canal, and improperly coiled cochlea (Hatch et al., 2007; Mansour et al., 1993). Further studies have shown a spectrum of phenotypic features in the inner ear of the FGF3 knockout animal models, which suggests the variable expressivity of *FGF3* mutations (Hatch et al., 2007; Kettunen et al., 2000). Moreover, *FGF10* and *FGF3* have been repeatedly shown to have an overlapping expression pattern in the otic placode, suggesting a modifier role for the *FGF10* gene in LAMM syndrome (Olaya‐Sánchez et al., 2017).

**FIGURE 3:**
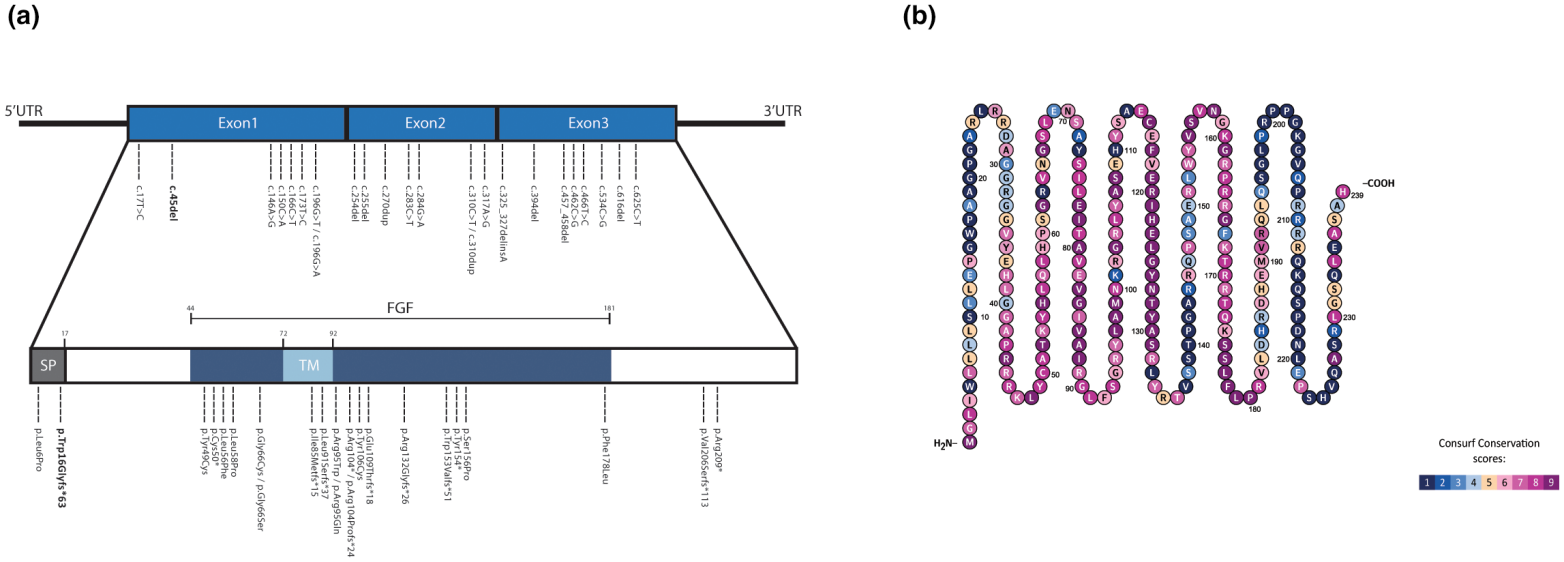
So far, 23 mutations spanning the FGF3 gene have been associated with labyrinthine aplasia, microtia, and microdontia syndrome. The FGF3 protein consists of one highly conserved fibroblast growth factor (FGF) domain (amino acids 44–181), a predicted signal peptide (SP, amino acids 1–17), and a predicted transmembrane domain (TM, amino acids 72–92) (a). The conservation scores of each individual amino acid were calculated using the ConSurf server (Ashkenazy et al., 2016). Note the high conservation scores for amino acids located in the FGF3 domain (b).

In humans, the *FGF7* subfamily is widely expressed in the mesenchymal tissues. Structural analysis shows that the members of the FGF7 subfamily (including FGF3, FGF10, FGF7, and FGF22) specifically bind to the b isoform forms of fibroblast growth factor receptors 1 and 2 (FGFR1b and FGFR2b) (Zinkle & Mohammadi, 2019). In addition to *FGF3*, mutations in the *FGF10* and *FGFR2b* have been associated with a syndromic form of deafness known as lacrimo‐auriculo‐dento‐digital (LADD) syndrome (OMIM 149730). Among the phenotypic features of the LADD syndrome, anomalies of the inner ear, outer ear, and teeth resemble those of LAMM syndrome (Ryu et al., 2020). Members of the FGF7 subfamily share a highly conserved 120–130 amino acid long fibroblast growth factor domain (Figure 3b), which is functionally essential for the FGF proteins (Zinkle & Mohammadi, 2019). In addition, the FGF3 protein is predicted to contain an N‐terminal signal peptide (phobius – amino acids 1–17) with a predicted cleavage site between amino acids 17 and 18 (https://services.healthtech.dtu.dk/service.php?SignalP), and a 20‐amino‐acid‐long transmembrane domain (phobius – amino acids 72–92). Signal peptide and transmembrane domains have been known to be essential for post‐translational modification in the endoplasmic reticulum; moreover, when deleting the signal peptide of the FGF3 protein, researchers observed the accumulation of the unmodified protein in the nucleus (Antoine et al., 1997). Therefore, destabilizing mutations in the signal peptide and transmembrane domain are predicted to reduce the secretion of the FGF3 protein and ultimately lead to reduced or complete loss of function of the protein.

Loss of function of the FGF3 protein, as indicated by animal studies, is responsible for symptoms of LAMM syndrome. Concordantly, almost half of the reported pathogenic and likely pathogenic variants (52%) are frameshift and stop‐gain variants that can either lead to the production of a truncated protein or nonsense‐mediated decay of the FGF3 transcript. Ten of the 11 remaining missense variants are located in the highly conserved fibroblast growth factor domain and one is within the secretory signal sequence (Figure 3a). Genotype–phenotype correlation of LAMM syndrome has not been thoroughly understood; however, it has been reported that individuals with homozygous p.R95W mutation had a less severe phenotype (Riazuddin et al., 2011). Moreover, Riazuddin et al. suggested that the p.R95W mutation had a semi‐dominant effect, as some carriers had mild to moderate bilateral conductive HL. Therefore, further investigations are needed stablish the phenotype–genotype association of LAMM syndrome.

In our study, we identified a novel mutation in the *FGF3* gene in a consanguineous Iranian family with three affected members which had identical LAMM syndrome features including HL, outer ear and teeth malformation. The HL was identified at birth in all three affected members; however, patients were not available to confirm inner ear malformation through further clinical testing. Neither of the discussed phenotypes were observed in the healthy siblings and the parents. Furthermore, our study strives to show that considering the specific facial (outer ear and teeth malformation) and clinical features (early onset severe to profound HL) of LAMM syndrome, it is suggested that in presence of such symptoms, the sequence of the *FGF3* gene be assessed in order to reduce the cost of genetic diagnostic measures.

## 5 CONCLUSION

Here, using WES, we introduce c.45delC as a novel mutation in the *FGF3* gene that co‐segregated with LAMM syndrome in a consanguineous Iranian family. This study is the first reported case of LAMM syndrome in Iran, and our data further broaden the spectrum of *FGF3* gene mutations.

## AUTHOR CONTRIBUTIONS

Fereshteh Jamshidi and Ebrahim Shokouhian: Data analysis and interpretation, drafting the article. Marzieh Mohseni: Data analysis and interpretation. Kimia Kahrizi: Clinical evaluation of family. Hossein Najmabadi: Design and implementation of the research, final approval of the version to be published. Mojgan Babanejad: Design and implementation of the research, final approval of the version to be published.

## FUNDING INFORMATION

This study was supported by University of Social Welfare and Rehabilitation Sciences, Grant Number: 2020‐T‐2405 to MB.

## CONFLICT OF INTEREST STATEMENT

The authors declare no conflict of interest.

## ETHICS APPROVAL STATEMENT

The procedure used in this study was reviewed and approved by the University of Social Welfare and Rehabilitation Sciences ethics committee.

## PATIENT CONSENT STATEMENT

Written consent was received from legal guardian of the patients.

## Data Availability

The datasets that support the findings of this study are available from the corresponding author upon reasonable request
